# Binocular Rivalry and Fusion-Inspired Hierarchical Complementary Ensemble for No-Reference Stereoscopic Image Quality Assessment

**DOI:** 10.3390/s26030883

**Published:** 2026-01-29

**Authors:** Yiling Tang, Shunliang Jiang, Shaoping Xu, Jian Xiao, Haiwen Yu

**Affiliations:** School of Mathematics and Computer Sciences, Nanchang University, Nanchang 330031, China; jiangshunliang@ncu.edu.cn (S.J.); xushaoping@ncu.edu.cn (S.X.); xiaojian@ncu.edu.cn (J.X.); yuhaiwen@ncu.edu.cn (H.Y.)

**Keywords:** stereoscopic image quality assessment, binocular rivalry, binocular fusion, multi-stage complementary ensemble, asymmetric distortion, Swin Transformer

## Abstract

No-reference stereoscopic image quality assessment (NR-SIQA) remains a fundamental challenge due to the complex biological mechanisms of binocular rivalry and fusion, particularly under asymmetric distortions. In this paper, we propose a novel framework termed Multi-Stage Complementary Ensemble (MSCE). The core innovation lies in the Adaptive Selective Propagation (ASP) strategy embedded within a hierarchical Transformer architecture to dynamically regulates the fusion of binocular features. Specifically, by simulating the human visual system’s transition from binocular rivalry to fusion, the ASP strategy applies nonlinear gain control to selectively reinforce features from the governing view based on binocular discrepancies. Furthermore, the proposed Hierarchical Complementary Fusion (HCF) module effectively captures and integrates low-level texture integrity, mid-level structural degradation, and high-level semantic consistency, leveraging ensemble learning principles, within a unified quality-aware manifold. Experimental results on four benchmark datasets demonstrate that the MSCE framework achieves state-of-the-art performance, particularly in terms of prediction consistency under complex asymmetric distortions.

## 1. Introduction

With the rapid deployment of immersive media systems, including virtual reality (VR) [1,2] and stereoscopic 3D displays, objective stereoscopic image quality assessment (SIQA) has become a foundational component in modern 3D imaging pipelines [3,4]. In contrast to conventional 2D image quality assessment (IQA) [5], SIQA requires explicit modeling of binocular interactions that govern human depth perception, perceptual stability, and visual comfort. In practical acquisition, compression, and transmission scenarios, stereoscopic content is frequently corrupted by asymmetric distortions, where the left and right views exhibit unequal degradation levels due to view-dependent noise, compression imbalance, or packet loss [6]. Under such conditions, directly aggregating monocular quality scores is insufficient for accurately predicting perceived visual quality, as binocular perception arises from complex mechanisms of binocular interactions and fusion rather than the independent evaluation of each view. Therefore, the development of SIQA models that can effectively reflect human visual perception remains an important yet challenging research problem. Moreover, given the high cost of collecting subjective data for stereoscopic content, the scarcity of labeled samples necessitates the development of data-efficient modeling approaches [7] that can map complex distortion manifolds to perceptual scores effectively.

Existing SIQA methods are commonly categorized into full-reference (FR), reduced-reference (RR), and no-reference (NR) approaches. Among these categories, NR-SIQA has attracted considerable attention for practical scenarios such as streaming and broadcasting, where reference images are generally unavailable. Early NR-SIQA methods evaluated the left and right views independently and combined their quality scores using simple averaging or fixed weighting strategies [8,9]. However, such approaches largely overlook binocular perceptual mechanisms and tend to suffer noticeable performance degradation under asymmetric distortions. To address this issue, subsequent studies attempted to model various aspects of binocular rivalry and fusion, which are fundamental mechanisms of binocular visual perception, in which asymmetrically distorted visual inputs presented to the left and right eyes alternately dominate perception [10,11,12]. These methods generally leverage the principles of binocular rivalry to fuse the left and right views into a single representation, from which low-level statistical features are extracted for quality assessment [13,14,15]. Furthermore, some works have utilized binocular summation and difference signals to better characterize distortion effects induced by asymmetric degradations across stereo pairs [16]. Although these handcrafted, binocular rivalry-inspired models provide certain performance gains, their dependence on static features and predefined fusion rules limits their robustness against complex asymmetric distortions.

Recent advances in deep learning have significantly improved SIQA performance. Early convolutional neural network (CNN)-based approaches adopt dual-stream architectures to extract monocular representations and perform fusion at high-level feature layers [17,18,19]. Despite their effectiveness, CNN-based models are inherently limited by their local receptive fields. This restriction hampers their ability to capture global structural degradation and long-range dependencies. More critically, these models typically rely on fixed, single-stage fusion strategies, which lack the flexibility to adapt to varying distortion levels or the reliability of different views. In recent years, substantial efforts have been devoted to enriching binocular fusion through attention mechanisms [20,21], multi-scale representations, and hierarchical fusion modules [22,23], leading to notable performance improvements in SIQA. Nevertheless, these approaches still face challenges in simulating complex binocular rivalry and fusion mechanisms. This is especially evident when modeling the dynamic and hierarchical nature of binocular integration, a process in the human visual system (HVS) that spans multiple visual processing levels, from early cortical areas to higher-order regions [21,24].

Vision Transformer (ViT) [25], particularly hierarchical variants such as Swin Transformer [26], offers a compelling alternative for modeling multi-stage visual processing. By leveraging self-attention within shifted windows, the Swin Transformer captures local detail degradation and global contextual distortions in a multi-scale manner. This capability has enabled transformer-based models to achieve state-of-the-art performance in 2D image quality assessment (IQA) recently, as demonstrated by TRIQ [27,28] and SwinIQA [29]. While recent surveys [30] confirm the effectiveness of this paradigm, the field continues to evolve with advanced strategies such as supervised contrastive learning [31] and efficient local feature adaptation [32]. Despite their success in 2D IQA, only a limited number of studies have explored the application of transformer-based architectures to SIQA. Effectively adapting these multi-scale attention-based models to SIQA by incorporating strategies that reflect the rivalry and hierarchical nature of binocular visual perception remains an open challenge and is critical for improving prediction accuracy.

Motivated by the biological mechanisms of binocular rivalry and fusion, alongside the hierarchical organization of the Human Visual System (HVS), we propose a novel NR-SIQA framework termed Multi-Stage Complementary Ensemble (MSCE). Built upon a Swin Transformer (SwinT) [26] backbone, MSCE implements an adaptive integration process that synthesizes quality-aware features across hierarchical stages. The efficacy of MSCE derives from the synergy between the Adaptive Selective Propagation (ASP) strategy and the Hierarchical Complementary Fusion (HCF) module. This approach explicitly models complex HVS interactions by integrating visual representations across multiple levels, spanning from low-level textures to high-level semantics.

1. We propose the Adaptive Selective Propagation (ASP) strategy, which models the dynamic transition from binocular rivalry to fusion. Through a stage-wise “sharpen-then-smooth” propagation, this strategy applies nonlinear gain control to adaptively modulate the perceptual influence of each view based on binocular discrepancies.
2. We develop the Hierarchical Complementary Fusion (HCF) module to preserve the integrity of multi-level feature representations. By maintaining three independent pathways, the module ensures that quality-aware cues from different abstraction levels are complementarily integrated without mutual interference, reflecting the parallel and hierarchical nature of biological visual processing.
3. We establish a unified SIQA framework that couples entropy-based statistical priors with deep hierarchical features. This hybrid design effectively balances the sensitivity to local structural degradations with global perceptual stability, achieving state-of-the-art performance across diverse distortion scenarios.

## 2. Related Work

Early NR-SIQA methods primarily relied on handcrafted features extracted independently from the left and right views, followed by conventional fusion strategies. Representative approaches computed statistical descriptors from each view and combined them through simple averaging or fixed weighting rules [8,9]. While computationally efficient, these methods fail to model binocular interactions and show a noticeable decrease in accuracy when evaluated on asymmetric distortions, where the distortion characteristics differ between the two views.

To better align with human visual perception and address the challenge of asymmetric distortions, subsequent studies incorporated mechanisms inspired by binocular rivalry and dominant-eye perception. Focusing specifically on asymmetric degradation, Wang et al. [33] investigated the quality assessment of asymmetrically compressed stereoscopic 3D videos, while Shao et al. [34] proposed a domain transfer framework to enhance prediction robustness across varying distortion asymmetries. Building on these insights, Fang et al. [16] utilized visual binocular properties, in particular binocular summation and difference statistics, to predict quality. Shen et al. [35] combined global and local content characteristics to capture varying distortion granularities.

Recent deep learning approaches have further advanced this direction by moving beyond independent view processing toward more sophisticated modeling of binocular interactions. Zhou et al. [1] were among the early explorations of interactive dual-stream networks in this field. Chang et al. [36] recently proposed a bidirectional feature aggregation network that utilizes parallax attention to enhance binocular fusion. Several studies have explicitly incorporated specific biological properties: Xu et al. [12] modeled the competitive nature of binocular rivalry using a predictive auto-encoding network, while Chang et al. [20] proposed a coarse-to-fine framework that leverages feedback guidance to simulate dominant eye perception. To capture comprehensive 3D information, researchers have also integrated supplementary visual cues. For instance, Li et al. [19] combined 3D visual saliency maps with CNN features, Sim et al. [37] explored the integration of binocular semantic and quality channels, and Shen et al. [38] fused 2D visual features with depth perception cues to address geometric inconsistencies. More structural approaches, such as the hierarchical multi-scale model by Chang et al. [23] and the parallel perception framework by Zhang and Li [22], aggregate features from different receptive fields to enhance robustness. Similarly, Messai and Chetouani [39] proposed an end-to-end multi-score model to predict quality at different stages of processing. Most recently, attention mechanisms have been employed to achieve adaptive binocular weighting. Wang et al. [40] introduced a binocular collaboration network that uses attention to weigh the contribution of each view dynamically. Li et al. [21] further advanced this by proposing a top-down stereo attention mechanism to guide quality prediction. While these methods can effectively improve the consistency between the estimated quality scores and human perception, further improvements are needed for the prediction of asymmetrically distorted images.

Beyond specific network architectures, researchers have also investigated diverse learning strategies and feature aggregation techniques to bolster model generalizability and robustness. To address complex distortion manifolds with limited data, Su et al. [7] proposed a data-efficient modeling approach. Rehman et al. [41] introduced cascaded networks to refine quality assessment for specific tasks like super-resolution, while Ahmed et al. [42] utilized ensemble strategies to stabilize predictions across varied degradation types. Regarding efficient regression backbones, the paradigm of mapping pooled features to quality scores remains a robust baseline. Varga demonstrated the efficacy of pooling deep representations, ranging from temporal pooling with SVR in video models [43] to advanced multiple pooling strategies in static images [44]. Recently, Hu et al. [45] further validated this direction by leveraging global awareness mechanisms to enhance feature representativeness.

During the past several years, Transformers have emerged as powerful alternatives to Convolutional Neural Networks for visual perception tasks, a shift comprehensively documented in recent surveys [30,46]. The Vision Transformer (ViT) [25] demonstrated that global self-attention can effectively capture long-range dependencies for image recognition. Building upon this paradigm, several IQA models using the Transformer architecture have been proposed. Cheon et al. introduced IQT [28], which leverages transformer encoders to model perceptual quality degradation. TRIQ [27] further refined this idea by learning quality aware representations through token interactions, and Shi et al. [31] enhanced feature discriminability via supervised contrastive learning. Hierarchical transformer architectures, such as Swin Transformer [26], have proven particularly effective for dense vision tasks due to their shifted window attention and multi-stage feature representation. Swin Transformer models for IQA, including SwinIQA [29], demonstrate strong performance on compressed image quality assessment by capturing both local artifacts and global structural distortions. Most recently, Xu et al. [32] proposed an efficient adaptation strategy to explicitly inject local distortion features into the Transformer backbone. To further refine feature integration, Yang and Li [47] proposed a multi-scale dual branch fusion network, while Zhang et al. [48] introduced information entropy to guide the Transformer, suggesting that statistical priors remain vital for attention modeling. These methods highlight the potential of hierarchical transformers for perceptual quality modeling.

However, applying successful strategies from 2D IQA to the stereoscopic domain remains non-trivial. Existing approaches typically process the left and right views independently and fuse their representations at a late stage, thereby overlooking the critical roles of binocular rivalry and binocular disparities. Furthermore, even among specialized SIQA models, binocular perception is often treated as a static integration process, failing to capture the dynamic rivalry that arises when views exhibit significant inconsistencies [11,33]. Additionally, while hierarchical feature extraction is widely employed [22,23], the distinct contributions of different abstraction levels to stereoscopic quality and the necessity for fusion weights at specific stages remain insufficiently explored. To address these limitations, we propose a biologically inspired framework that explicitly models the progressive nature of binocular rivalry and the multi-level complexity of stereoscopic perception.

## 3. Proposed Method

### 3.1. Motivation and Framework Overview

The human visual system (HVS) does not perceive stereoscopic images through a simple additive process. Instead, it involves a sophisticated interplay between binocular fusion and rivalry [20,21,33]. When monocular inputs are consistent, the brain achieves smooth integration; however, in the presence of asymmetric distortions, perception is often dominated by the view with higher information salience or better quality [10,33].

Inspired by these biological principles, we propose two core mechanisms to enhance SIQA performance. First, the ASP strategy functionally simulates the progressive nature of binocular rivalry. Unlike static weighting schemes, ASP dynamically models competitive interactions, allowing the model to adaptively shift focus between views across processing stages. This mimics the HVS’s ability to handle dynamic perceptual transitions; specifically, the nonlinear weight reinforcement in ASP Stage 1 is grounded in Stevens’ Power Law [49], simulating the discriminative selection behavior where perceptual response scales nonlinearly with conflict intensity. Second, the HCF module abstracts the HVS’s parallel neural pathways. Recognizing that low-level textures and high-level semantics contribute differently to quality, HCF employs independent fusion channels with stage-specific weights. This strategy, analogous to ensemble learning, ensures that complementary quality-aware attributes at various levels are preserved and integrated in a manner consistent with hierarchical visual processing.

It is important to clarify that ASP and HCF are functional abstractions rather than detailed biophysical simulations. While they capture essential perceptual behaviors such as rivalry dynamics and hierarchical fusion, they do not replicate the continuous adaptation, recurrent connectivity, or neuromodulatory dynamics of the biological visual system. To translate these biological insights into a computationally feasible model, we explicitly adopt several engineering approximations. For instance, we utilize the entropy of the Mean Subtracted Contrast Normalization (MSCN) coefficients [50] to capture local information complexity and perceptual uncertainty, avoiding the complexity of simulating stochastic neural spiking. Similarly, the hierarchy of three pathways serves as a discrete abstraction of the continuous processing across multiple layers of the cortex. Furthermore, a fixed backbone with shared weights based on the Swin Transformer is employed to ensure feature stability during training, which contrasts with the dynamic synaptic plasticity of biological networks. Regarding the mathematical implementation, the functional forms governing rivalry and fusion are chosen for their computational efficiency and are parameterized within perceptually plausible ranges derived from established psychophysical models. This hybrid approach allows the MSCE framework to leverage fundamental perceptual principles while remaining implementable for practical quality assessment tasks.

Building on these motivations, we introduce the MSCE framework to align deep learning architectures with the biological principles of binocular perception. MSCE formalizes the NR-SIQA task as a progressive and selective integration process, mapping a stereoscopic image pair $\left(I_{L},I_{R}\right)$ to a perceptual quality score *Q* through a hierarchy of quality-aware representations.

**Figure 1:**
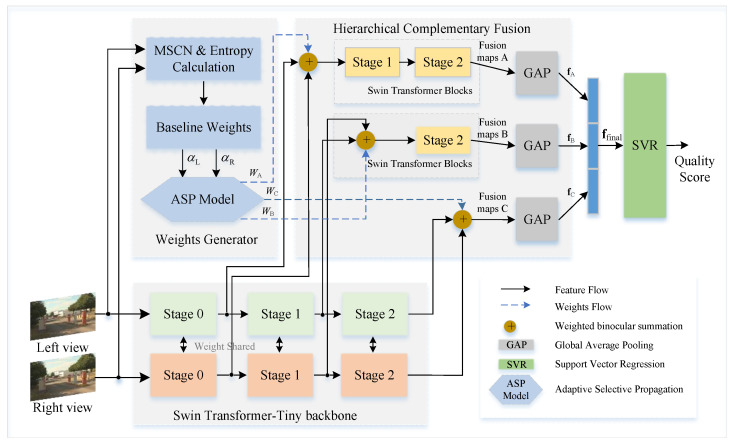
The overall architecture of the proposed Multi-Stage Complementary Ensemble (MSCE) framework. The system processes stereo views through a shared hierarchical backbone. Feature maps are subsequently integrated by the Hierarchical Complementary Fusion (HCF) module through three independent pathways (Paths A, B, and C). The fusion process is governed by the Adaptive Selective Propagation (ASP) strategy, which employs a nonlinear reinforcement strategy to refine binocular weights ($W_{A},W_{B},W_{C}$) derived from entropy-based priors. The fused features are aggregated to Global Average Pooling (GAP) and final SVR regression.

As illustrated in Figure 1, the proposed framework adopts a stage-wise processing pipeline consisting of three synergistic components. The input stereo image is first processed by a shared hierarchical backbone based on the Swin Transformer Tiny (SwinT) architecture [26]. By utilizing weight-sharing across sequential stages, the backbone extracts a range of multi-stage feature representations, capturing quality-aware cues ranging from detailed texture integrity to high-level semantic structures. This hierarchical design enables the model to adapt to diverse distortion types by incorporating features at multiple levels of abstraction.

The core of the integration process centers on the HCF module, which operates under the guidance of the ASP strategy. Rather than employing fusion at a single stage, the HCF component implements three independent pathways that correspond to the distinct computational stages of the backbone. This architecture ensures that complementary perceptual attributes are preserved without mutual interference. Within these pathways, the fusion process is dynamically regulated by the ASP strategy. By implementing a nonlinear reinforcement logic grounded in MSCN-based entropy statistics, ASP adaptively modulates binocular weights by characterizing binocular inconsistencies. This strategy ensures that features critical for quality discrimination from the dominant view are accentuated while the influence of heavily distorted counterparts is adaptively attenuated, effectively simulating the dynamic gain control of the HVS.

Finally, the fused features from the three independent pathways are concatenated into a global quality descriptor. This aggregated representation is subsequently mapped to the final perceptual quality score through SVR. By synthesizing these complementary features, the MSCE framework ensures a comprehensive evaluation that accounts for both local structural details and global semantic content. The strategy of extracting deep features from multiple layers and mapping them via Global Average Pooling (GAP) and SVR has been proven effective in several NR-IQA studies. For instance, Varga [43,44] demonstrated that multi-scale pooling of deep features can effectively capture varied distortion granularities. While our framework adopts a similar regression backbone, the core distinction lies in the introduction of the ASP strategy. This approach transcends such static aggregation by modeling the dynamic, rivalry-aware binocular interactions across hierarchical levels [6], moving beyond simple spatial scales to address the complex perception specific to stereoscopic vision.

### 3.2. Shared Multi-Level Feature Extraction

We utilize the SwinT [26] as the shared backbone for multi-level feature extraction. This architecture is selected for its shifted-window attention mechanism, which effectively captures both local structural artifacts and global semantic distortions. To ensure robustness and focus on validating the proposed binocular fusion mechanisms, the backbone is initialized with ImageNet pre-trained weights and serves as a fixed feature extractor without further fine-tuning. This strategy aligns with established transfer learning practices in IQA [28], where fixed pre-trained extractors provide a stable perceptual foundation while preventing overfitting on limited SIQA datasets.

The left and right views are processed through a shared backbone to generate hierarchical feature maps across three distinct levels. Formally, let $\Phi^{\left(s\right)}\left(\cdot \right)$ denote the feature extraction function up to stage s∈{0, 1, 2}, which yields the binocular feature pairs:

(1) $$F_{L}^{\left(s\right)}=\Phi^{\left(s\right)}\left(I_{L}\right),\;F_{R}^{\left(s\right)}=\Phi^{\left(s\right)}\left(I_{R}\right)$$

The resulting features are characterized by increasing levels of abstraction: (1) Low-level features (Stage 0), $F^{\left(0\right)}\in R^{\frac{H}{4}\times \frac{W}{4}\times C_{0}}$, capturing fine-grained texture and edge information; (2) Mid-level features (Stage 1), $F^{\left(1\right)}\in R^{\frac{H}{8}\times \frac{W}{8}\times C_{1}}$, representing structural components; (3) High-level features (Stage 2), $F^{\left(2\right)}\in R^{\frac{H}{16}\times \frac{W}{16}\times C_{2}}$, encoding global semantic consistency. These hierarchical representations serve as the multi-level basis for the subsequent ASP and fusion processes.

### 3.3. Global Binocular Weighting Baseline

To establish a statistical foundation for modeling binocular rivalry and fusion, we derive global priors to estimate the relative perceptual reliability of each view. This baseline weighting mechanism serves as the reference for subsequent stage-wise propagation. We first apply the MSCN transform [50] to extract structural information from local luminance. For a pixel (i,j), the normalized coefficient $\hat{I}\left(i,j\right)$ is defined as:

(2) $$\hat{I}\left(i,j\right)=\frac{I(i,j)-\mu (i,j)}{\sigma \left(i,j\right)+C_{c}}$$

where $C_{c}$ is a stability constant. The local mean μ(i,j) and standard deviation σ(i,j) are computed using a Gaussian window $\omega_{k,l}$:

(3) $$\mu \left(i,j\right)=\sum_{k=-K}^{K}\sum_{l=-L}^{L}\omega_{k,l}I\left(i+k,j+l\right)$$

(4) $$\sigma \left(i,j\right)=\sqrt{\sum_{k=-K}^{K}\sum_{l=-L}^{L}\omega_{k,l}{\left(I(i+k,j+l)-\mu (i,j)\right)}^{2}}$$

Since distortions typically disrupt the natural statistics of MSCN coefficients, we quantify this degradation through information entropy. The entropy *E* is defined as:

(5) $$E=-\sum_{v}p\left(v\right)\log_{2}p\left(v\right)$$

where p(v) represents the probability distribution of quantized MSCN coefficients. Given the entropies of the left and right views, $E_{L}$ and $E_{R}$, the baseline fusion weights $\left(\alpha_{L},\alpha_{R}\right)$ are formulated as:

(6) $$\alpha_{L}=\frac{E_{R}}{E_{L}+E_{R}},\;\alpha_{R}=\frac{E_{L}}{E_{L}+E_{R}}$$

This formulation assigns a higher weight to the view with lower statistical uncertainty, effectively identifying the dominant view that likely governs the initial stage of binocular rivalry. These priors grounded in entropy measures serve as a robust statistical foundation for the subsequent reinforcement of binocular rivalry within the ASP stage.

**Figure 2:**
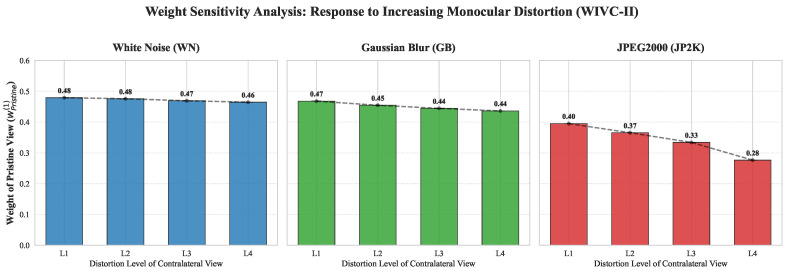
Variation of ASP weights across increasing monocular distortion levels in the WIVC-II [51] dataset. The different colors represent various distortion types, and the dashed lines denote the trend for each specific category. The y-axis represents the fusion weight assigned to the pristine view. The strictly monotonic decrease in the pristine view’s weight illustrates the distortion-governing mechanism, where severe monocular artifacts act as the dominant determinant of the overall quality.

To empirically validate the proposed weighting mechanism, we conduct a representative case study using samples from the Waterloo-IVC 3D Phase II (WIVC-II) [51] dataset. As illustrated in Figure 2, the weights assigned to the pristine view exhibit a monotonic decrease as monocular degradation intensifies. This trend reveals a distortion-governing behavior in binocular perception: rather than simply prioritizing the high-quality channel, the human visual system is acutely sensitive to significant monocular artifacts, which effectively become the dominant determinant of the overall viewing experience. The declining influence of the pristine view indicates that severe distortions in one channel can dominate the overall quality perception, overriding the contribution of the intact signal. This effect is particularly pronounced in structural degradations (GB and JP2K), where the weight converges toward the distorted channel to reflect the resulting perceptual collapse. As the distortion level increases, the weight shift toward the distorted channel demonstrates the linear relationship between the perceived quality and the degradation severity, aligning with the observed quality scores. The MSCN entropy-based weights thus ensure that the loss of structural integrity is fully captured in the feature representation, accurately reflecting the perceptual effects observed in human vision.

### 3.4. Adaptive Selective Propagation (ASP) Strategy

Although the entropy-based global weights $\left(\alpha_{L},\alpha_{R}\right)$ establish a robust global prior, applying them uniformly across all network stages ignores the dynamic nature of binocular integration. The HVS processes visual information hierarchically, where weak rivalry typically requires different integration strategies compared to strong rivalry. To simulate this physiological behavior, we propose the ASP strategy to deterministically propagate and reinforce the baseline weights across multi-level feature hierarchies. The ASP operates through three conceptual phases applied to the global rivalry intensity: Baseline Preservation, Rivalry-Aware Reinforcement, and Adaptive Smoothing. These adjusted weights directly govern the three parallel pathways within the HCF module, ensuring that quality-aware features are integrated through a “sharpen-then-smooth” logic: the weights are first intensified in Stage 1 to emphasize the dominant view, and subsequently regularized in Stage 2 to achieve a balanced and stable feature fusion.

We first define the binocular rivalry intensity *C* to quantify the deviation from equilibrium implied by the baseline weights:

(7) $$C=2\;\cdot \;|\alpha_{L}-0.5|=2\;\cdot \;|\alpha_{R}-0.5|,\;C\in \left[0,1\right]$$

The rivalry intensity *C*, derived from the global entropy priors, provides a stage-invariant measure of the competitive landscape, ranging from weak competition characterized by ambiguous dominance (C≈0) to to strong competition clear dominance (C≈1). Leveraging this consistent competitive context, the ASP strategy generates stage-specific fusion weights $w_{L}^{\left(s\right)},w_{R}^{\left(s\right)}$ for s∈0, 1, 2 through a structured three-phase process:

(1) Stage 0: Baseline Preservation. At the earliest stage, the system strictly adheres to the global statistical prior to maintain the fundamental quality assessment derived from MSCN entropy. This ensures that the low-level texture integrity in Path A is preserved based on the initial reliability estimate. The Stage 0 weights are defined as:

(8) $$w_{L}^{\left(0\right)}=\alpha_{L},\;w_{R}^{\left(0\right)}=\alpha_{R}$$

(2) Stage 1: Rivalry-Aware Reinforcement. Recognizing that mid-level features benefit from decision sharpening, we introduce a nonlinear reinforcement factor E(C) that modulates the perceptual gain according to the competitive landscape:

(9) $$E\left(C\right)=E_{\max}-\left(E_{\max}-E_{\min}\right)\cdot C^{\gamma }$$

The weights for Stage 1 are subsequently computed as:

(10) $$w_{L}^{\left(1\right)}=\mathrm{clamp}\left(0.5+E\left(C\right)\cdot \left(\alpha_{L}-0.5\right),\;0,\;1\right)$$

where clamp(x,a,b)=max(a,min(b,x)) ensures that the weights remain within the valid range [0, 1]. Based on extensive empirical validation, the hyperparameters are set to $E_{\min}=2.0,\;E_{\max}=4.0$, and the adaptivity factor γ=0.6. Crucially, setting $E_{\min}\geq 2.0$ introduces a significant nonlinear gain to reinforce the dominance of the leading view. The inclusion of the adaptivity factor γ optimizes the response curvature; our sensitivity analysis indicates that γ=0.6 provides high sensitivity to binocular disparities, effectively simulating the binocular rivalry mechanism. While more aggressive reinforcement (e.g., $E_{\max}=8.0,\;\gamma =1.0$) can further polarize weights in extreme asymmetric cases, our experiments reveal that the proposed configuration achieves an effective balance between enhancing the perceptual sensitivity to binocular rivalry and preserving the structural stability required for symmetric fusion.

(3) Stage 2: Adaptive Smoothing. At the deepest semantic level, we apply a smoothing factor S(C) to prevent excessive feature polarization in Path C, ensuring that global semantic consistency is maintained through perceptual integration:

(11) S(C)=1−(λ·γ)·C,whereλ=0.3

(12) $$w_{L}^{\left(2\right)}=\mathrm{clamp}\left(0.5+S\left(C\right)\cdot \left(w_{L}^{\left(1\right)}-0.5\right),0,1\right)$$

The hyperparameter λ is empirically set to 0.3 based on grid-search validation. This value strikes an optimal balance between the sharp reinforcement of monocular rivalry and the stable integration of binocular fusion features. The corresponding right-view weights are consistently defined as $w_{R}^{\left(s\right)}=1-w_{L}^{\left(s\right)}$.

The mathematical formulation in Equations (9)–(12) serves as a computational proxy for the nonlinear gain control observed in biological binocular rivalry. The reinforcement function E(C) simulates discriminative selection by incorporating a power law term $C^{\gamma }$. This design is motivated by Stevens’ Power Law ($\psi \propto I^{\gamma }$) [49], in which the perceived magnitude ψ corresponds to the feature reinforcement weight, and the physical stimulus intensity *I* corresponds to the contrast between views. This modeling choice is further supported by studies on visual contrast discrimination [52], which show that perceptual responses follow a power law relationship with stimulus intensity differences.

In the proposed model, the adaptivity factor γ regulates the curvature of the response function. Setting γ to 0.6 produces a convex profile in which the response gradient becomes steeper near the state of zero conflict, increasing sensitivity to subtle binocular discrepancies. As a result, even minor quality differences between the two views can trigger a clear shift toward the prioritized view. The bounds $E_{\min}$ and $E_{\max}$ define the dynamic range of the rivalry process, allowing the network to transition smoothly between discriminative selection and binocular fusion according to the clarity of the input signal.

**Figure 3:**
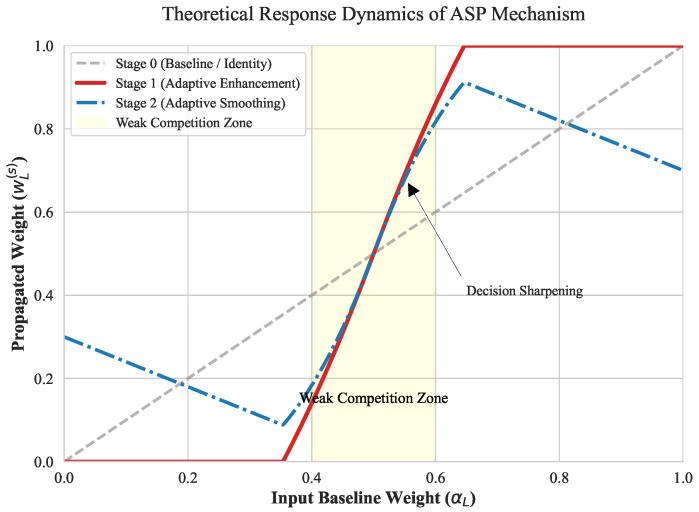
Theoretical response dynamics of the ASP strategy under optimized parameters ($E_{\min}=2.0$, $E_{\max}=4.0$). The curves illustrate the transition from aggressive decision sharpening in Stage 1 to adaptive smoothing in Stage 2.

To validate the proposed ASP strategy, we perform a detailed analysis of its response dynamics. As illustrated in Figure 3, the mapping functions from the baseline entropy weight $\alpha_{L}$ to the propagated weights reveal three critical behaviors. In the “Weak Competition” zone ($\alpha_{L}\in \left[0.4,\;0.6\right]$), the Stage 1 curve (red solid line) exhibits a highly aggressive gradient compared to the Stage 0 baseline. This intensified nonlinear effect confirms that ASP performs decision sharpening, effectively amplifying subtle inter-view quality differences to facilitate the identification of dominant visual cues. As the input weight $\alpha_{L}$ moves toward the extrema, the enhancement effect quickly reaches saturation. This ensures that for structurally evident asymmetric distortions, the strategy effectively reinforces the perceived dominance of the superior view by adhering to the established statistical priors. Furthermore, the Stage 2 curve (blue dash-dotted line) demonstrates an adaptive smoothing behavior, residing between the sharpened Stage 1 output and the baseline to provide essential regularization. This multi-stage evolution prevents over-fitting to noisy estimations and ensures a stable optimization landscape.

**Figure 4:**
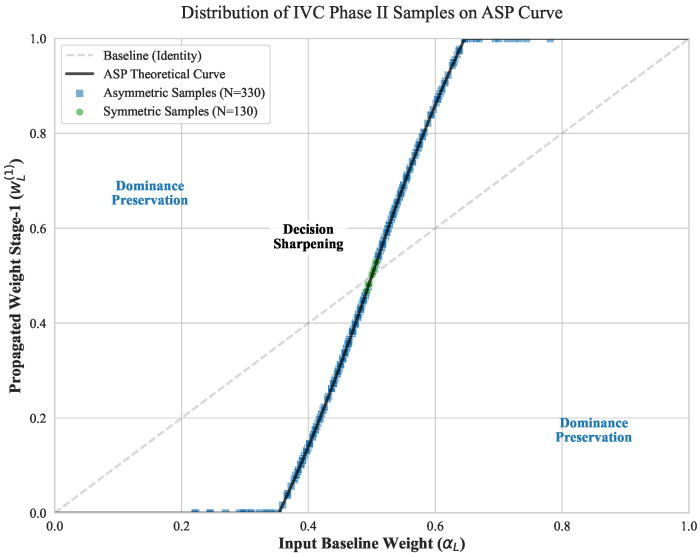
Empirical verification of the ASP strategy on the WIVC-II dataset. The plot illustrates the distribution of samples on the Stage 1 ($w_{L}^{\left(1\right)}$) response curve. Green circles denote symmetric samples (N=130), while blue squares denote asymmetric samples (N=330).

To verify this strategy in real-world scenarios, we visualized the weight distribution after Stage 1 propagation ($w_{L}^{\left(1\right)}$) for 460 image pairs from the WIVC-II dataset. As shown in Figure 4, the empirical data distribution closely aligns with these optimized response curves. First, symmetric samples (green circles, N=130) are accurately captured by the steepest region of the gradient, confirming active decision sharpening for ambiguous inputs. Second, asymmetric samples (blue squares, N=330) cluster in the dominance preservation zones. This data-driven evidence suggests that the proposed ASP module effectively switches between sharpening and dominance-preservation modes in accordance with the competitive intensity of realistic stereoscopic content.

To further investigate the internal dynamics of the ASP strategy, we conduct a representative case study using samples from the WIVC-II [51] dataset. To isolate the impact of distortion-driven competition, all selected samples originate from the same scene (“CraftLoom”), covering symmetric and various asymmetric scenarios.

The results in Table 1 confirm the precision of the ASP strategy. For the symmetric sample (G2–G2), the weights remain near 0.5, indicating that the scheme maintains integrative consistency when competition is balanced. In contrast, for the asymmetric case (Re–J2), where the disparity between views is significant, the weights are nonlinearly modulated to capture the impact of the distorted channel. In Stage 1, the weight for the pristine view $\alpha_{L}$ is reduced from 0.4021 to 0.1821, reflecting the increased perceptual dominance of the distorted right view (J2) in anchoring the overall quality. In Stage 2, this is followed by a slight smoothing adjustment to 0.1933 for the pristine view, which ensures numerical stability while maintaining the distorted signal as the primary determinant of the final score. For cases with subtle binocular disparity (e.g., W2–W4), the strategy effectively sharpens the influence of the perceptually dominant view. This stage-wise evolution validates our proposed “sharpen-then-smooth” logic, where the initial reinforcement of competitive signals is followed by an adaptive smoothing process, ensuring robust feature integration across diverse distortion landscapes.

### 3.5. Hierarchical Complementary Fusion (HCF)

The HCF module serves as the structural core of the MSCE framework, designed to integrate binocular information across three independent and parallel pathways. By modeling the fusion process into stage-wise operations, HCF ensures that quality-aware features at different abstraction levels are preserved and modulated without mutual interference. Crucially, rather than relying on static aggregation, the HCF module operates under the deterministic guidance of the ASP-refined weights $w_{L}^{\left(s\right)},\;w_{R}^{\left(s\right)}$, which adaptively govern the contribution of each view based on the competitive landscape.

These three pathways correspond directly to the ASP conceptual phases: Path A implements the baseline preservation phase by integrating Stage 0 features to preserve low-level texture integrity using the baseline weights $w^{\left(0\right)}$; Path B realizes the competition-aware reinforcement phase by fusing Stage 1 features guided by the nonlinearly reinforced weights $w^{\left(1\right)}$ to model the binocular rivalry mechanism, where dominant structural cues are adaptively accentuated through a selective dominance logic to ensure that quality-discriminative information prevails over distorted counterparts; and Path C executes the adaptive smoothing phase by merging Stage 2 semantic representations using the regularized weights $w^{\left(2\right)}$ to maintain global scene consistency through integrative consistency. The fused feature map for each stage s∈0, 1, 2 is formally expressed as:

(13) $$F_{\mathrm{fused}}^{\left(s\right)}=w_{L}^{\left(s\right)}\cdot F_{L}^{\left(s\right)}+w_{R}^{\left(s\right)}\cdot F_{R}^{\left(s\right)}.$$

To further optimize these initial representations, each pathway incorporates a complementary feature refinement stage consisting of residual convolutional layers and a local attention strategy. This unit re-calibrates the feature response $F_{\mathrm{fused}}^{\left(s\right)}$ based on the stage-specific receptive field to extract discriminative quality cues while mitigating potential fusion inconsistencies. The resulting refined maps ${\hat{F}}^{\left(s\right)}$ are then transformed into compact global descriptors $f_{A},f_{B},f_{C}\in R^{384}$ via GAP:

(14) $$f_{k}=\mathrm{GAP}\left({\hat{F}}_{k}\right),\;k\in \left\{A,B,C\right\}.$$

Finally, these descriptors are concatenated to form the integrated quality-aware representation:

(15) $$f_{\mathrm{final}}=\left[f_{A}⊕f_{B}⊕f_{C}\right]\in R^{1152}.$$

This multi-stage ensemble vector encapsulates a wide spectrum of binocular interactions, ranging from coherent fusion in symmetric scenarios to the selective dominance induced by asymmetric rivalry. The final quality score *Q* is regressed through an SVR module with a Radial Basis Function (RBF) kernel. The SVR effectively models the nonlinear relationship between multi-stage features and human subjective perception, while ensuring robust generalization on the typically small-sample datasets characteristic of SIQA tasks.

## 4. Experimental Results and Analysis

### 4.1. Datasets and Experimental Setup

**Figure 5:**
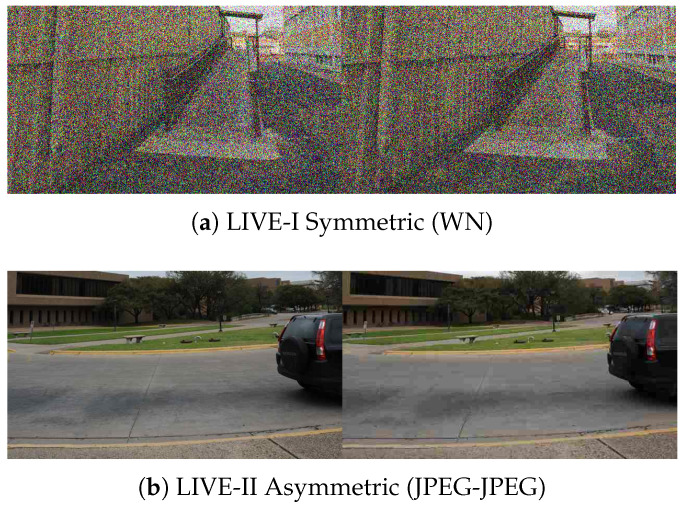

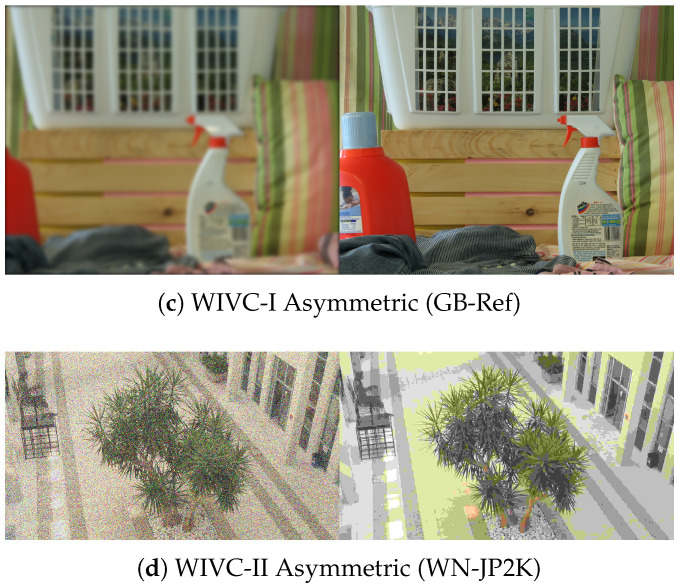
Representative stereoscopic image pairs from the benchmark datasets illustrating the progressive complexity of binocular distortions. (**a**) A sample from LIVE-I with symmetric White Noise distortion. (**b**) A sample from LIVE-II with asymmetric JPEG compression. (**c**) A sample from WIVC-I exhibiting single-view distortion. (**d**) A sample from WIVC-II showing complex mixed-type asymmetric distortion, which triggers intense binocular rivalry and necessitates the proposed ASP strategy.

To provide a comprehensive assessment of model generalization, we evaluate our method on four benchmark stereoscopic image quality assessment (SIQA) datasets: LIVE 3D Phase I (LIVE-I) [13], LIVE 3D Phase II (LIVE-II) [53], Waterloo-IVC 3D Phase I (WIVC-I) [10], and WIVC-II [51]. These datasets collectively offer a diverse range of distortion types, including JPEG Compression (JPEG), JPEG2000 (JP2K), White Noise (WN), Gaussian Blur (GB), and Fast Fading (FF), spanning a wide spectrum of binocular symmetry levels. Specifically, the LIVE-I dataset contains only symmetrically distorted pairs, providing a baseline for balanced binocular viewing conditions. In contrast, the remaining three datasets are hybrid collections incorporating both symmetric and asymmetric scenarios. LIVE-II primarily introduces asymmetric configurations where both views suffer from the same distortion type at different intensity levels. The WIVC-I and WIVC-II datasets feature more challenging asymmetric configurations where the left and right views may encounter entirely different distortion types or single-view distortions. Subjective quality labels are provided as Difference Mean Opinion Scores (DMOS) for the LIVE series and Mean Opinion Scores (MOS) for the WIVC series. Representative stereoscopic pairs from these datasets, illustrating the varying complexity of binocular distortions, are presented in Figure 5.

The proposed MSCE framework is implemented using the PyTorch (https://pytorch.org, accessed on 26 January 2026) library. To ensure the robustness and generalization of feature representation, we utilize the official Swin-Tiny backbone as a fixed feature extractor without additional fine-tuning. This backbone is pre-trained on the ImageNet-1K dataset. Multi-level features are extracted from the hierarchical stages of the Transformer and subsequently processed through the ASP and HCF modules to generate the final quality-aware feature vectors. All experiments are conducted on a workstation equipped with an Intel Core i7-11700 CPU and an NVIDIA RTX A4000 GPU.

The quality regression stage is performed using Support Vector Regression (SVR) with a radial basis function (RBF) kernel. To ensure the statistical reliability of the performance metrics, we implement a rigorous evaluation protocol consisting of 5 independent trials. In each trial, a 10-fold cross-validation procedure is conducted by randomly shuffling and splitting the dataset. The SVR hyperparameters are kept constant across all experiments, with the penalty parameter *C*, the kernel coefficient $\gamma_{\mathrm{svr}}$, and the tube size ϵ set to 512, 0.0039, and 1.0, respectively. By maintaining consistent SVR settings and keeping the backbone parameters fixed, we ensure that the performance improvements are strictly attributable to the proposed multi-level feature integration design.

### 4.2. Comparison with State-of-the-Art Methods

To demonstrate the effectiveness of the proposed MSCE framework, we conduct a comparative analysis against a set of state-of-the-art methods. Comparative results are obtained from original publications under consistent dataset configurations. The comparison group encompasses traditional approaches, including two FR metrics, ChenFR [13] and IDWSSIM [10], as well as three NR metrics based on natural scene statistics, BRISQUE [50], ChenNR [14], and Shen [38]. Furthermore, to reflect recent advancements in deep learning, we compare our method with several CNN-based SIQA approaches, including Liu [54], Si [6], Sim [37], X-Net [40], and Li [21].

The experimental results on the WIVC and LIVE datasets are summarized in Table 2 and Table 3. Notably, our method achieves state-of-the-art performance across most metrics. To further verify the reliability of these improvements, we conducted paired *t*-tests (at 95% confidence level) between the SROCC/PLCC distributions of our MSCE framework and the competing methods over 5 independent runs. The results marked with an asterisk (*) indicate statistically significant superiority (p<0.05). Specifically, on the challenging WIVC-II dataset, MSCE yields a PLCC of 0.984 and an SROCC of 0.983, significantly outperforming existing models with p<0.001. On LIVE-II, while the correlation metrics are competitive with X-Net, our method achieves a substantially lower RMSE (2.752 vs. 5.029), demonstrating superior prediction stability.

Furthermore, Table 3 presents the results on the LIVE-I and LIVE-II datasets. On the symmetric LIVE-I dataset, MSCE maintains competitive performance, demonstrating that the inclusion of rivalry-aware modules does not compromise the model’s ability to handle symmetric distortions. On LIVE-II, MSCE achieves a notable RMSE of 2.752, representing the highest prediction accuracy among all compared methods. The consistent superiority of MSCE across datasets with varying distortion characteristics confirms its robustness and generalization capability.

### 4.3. Performance Analysis by Distortion Type

To verify the robustness of the proposed framework against specific degradation patterns, we analyze the performance on the LIVE-I and LIVE-II datasets categorized by individual distortion types: JP2K, JPEG, WN, GB, and FF. Table 4 and Table 5 summarize the SROCC and PLCC results for these categories. While some optimized CNN models (e.g., Li et al. [21] and X-Net [40]) exhibit high performance on specific distortion types such as WN or GB, the proposed MSCE maintains consistently competitive and stable results across all categories without dramatic performance fluctuations. Specifically, on the LIVE-I dataset, MSCE achieves an SROCC of 0.971 for WN, matching the third-best performance, and maintains high reliability (>0.92) across GB and FF. On the LIVE-II dataset, which contains asymmetric samples, MSCE demonstrates superior robustness by achieving the second-best SROCC across four out of five categories (JP2K, WN, GB, and FF). The high PLCC values on LIVE-II (e.g., 0.993 for GB and 0.974 for WN) further indicate that the MSCE framework effectively captures feature responses to handle binocular rivalry, even when the distortion characteristics vary significantly. This balanced performance across diverse scenarios, rather than overfitting to a single distortion type, underscores the generalization capability of MSCE as a comprehensive quality evaluator.

### 4.4. Performance on Symmetric vs. Asymmetric Distortions

To further investigate the effectiveness of the MSCE framework, particularly its ability to handle binocular rivalry, we conduct a detailed performance analysis on the WIVC-I, WIVC-II, and LIVE-II datasets. The primary objective is to verify whether the model maintains robustness under varying degrees of inter-view consistency. Accordingly, the test samples in each dataset are categorized into symmetric and asymmetric subsets based on the distortion configurations of the left and right views. This stratification facilitates a detailed examination of how different fusion strategies respond to varying levels of binocular competition. Table 6 summarizes the performance metrics (SROCC, PLCC, and RMSE) for the overall, symmetric, and asymmetric partitions.

The results in Table 6 indicate that MSCE achieves high correlation scores across both subsets. Notably, on the WIVC-II dataset that contains complex rivalry scenarios, the performance on the asymmetric subset (SROCC 0.9824, PLCC 0.9836) is nearly equivalent to the symmetric subset (SROCC 0.9832, PLCC 0.9845). This high level of consistency underscores the effectiveness of the ASP strategy in adaptively modulating feature weights when quality imbalance occurs between the two views. Furthermore, the stable RMSE across both partitions confirms that the MSCE framework effectively mitigates the prediction variance induced by binocular rivalry, which establishes a reliable mapping from latent stereoscopic features to subjective quality scores.

### 4.5. Cross-Dataset Generalization Ability

To evaluate the generalization capability of the proposed MSCE framework, we conduct cross-dataset evaluations where the model is trained on a source dataset and directly tested on an unseen target dataset without any fine-tuning. We follow the standard Test/Train notation to denote each experimental configuration. This setup poses a significant challenge due to the substantial domain shift in terms of distortion types, image content, and the distribution of subjective scores. Table 7 summarizes the SROCC and PLCC results in comparison with several state-of-the-art methods.

The experimental results demonstrate the robustness of the MSCE framework across different domains. Regarding the WIVC benchmarks, the proposed method achieves an SROCC of 0.922 and a PLCC of 0.930 in the WIVC-I/WIVC-II scenario. This indicates that the perceptual features extracted from asymmetric distortions generalize effectively to symmetric scenarios, confirming the versatility of the multi-stage fusion strategy. In the LIVE cross-dataset tests, the proposed method exhibits superior stability. Specifically, for the LIVE-I/LIVE-II case, our approach yields an SROCC of 0.885, which outperforms the recent method by Li [21] (0.855). This margin suggests that the entropy-based ASP strategy captures intrinsic quality-aware cues that are less susceptible to dataset-specific biases. While the method by Chang [20] achieves slightly higher performance in specific configurations, the MSCE framework maintains a highly competitive and balanced performance across all testing pairs. These results collectively confirm that the stage-wise propagation and entropy-based weighting possess strong universal applicability across diverse stereoscopic scenarios.

### 4.6. Ablation Study

To validate the architectural design of the MSCE framework and systematically isolate the contributions of its individual components, we conduct a comprehensive series of ablation experiments.

#### 4.6.1. Impact of Regression Head Selection: SVR vs. MLP

The employment of fully connected layers or Multi-Layer Perceptrons (MLP) as regression heads has been widely adopted in deep learning-based IQA, demonstrating remarkable success in large-scale visual recognition and quality assessment tasks. To evaluate the applicability of these standard architectures within the context of SIQA, which typically involves smaller-scale datasets, we designed two MLP configurations, inspired by recent state-of-the-art studies [21,40], to compare with the proposed SVR scheme. Specifically, the first variant is a single-hidden-layer MLP configured as 1152→256→1, utilizing ReLU activation and a dropout rate of 0.5. The second is a deeper dual-hidden-layer variant structured as 1152→512→128→1, which incorporates cascading dropout layers (0.5 and 0.3) to mitigate overfitting. We compared the performance of our SVR-based method with these two MLP regression heads. To ensure optimal convergence for varying depths, we tailored the training protocols for each variant over 300 epochs: the Single-Layer MLP was trained with a larger batch size of 128 and a learning rate of $10^{-3}$ (weight decay $10^{-4}$), whereas the deeper Double-Layer model required a more conservative optimization strategy with a batch size of 64 and a reduced learning rate of $10^{-4}$ (weight decay $10^{-5}$) to maintain stability.

**Figure 6:**
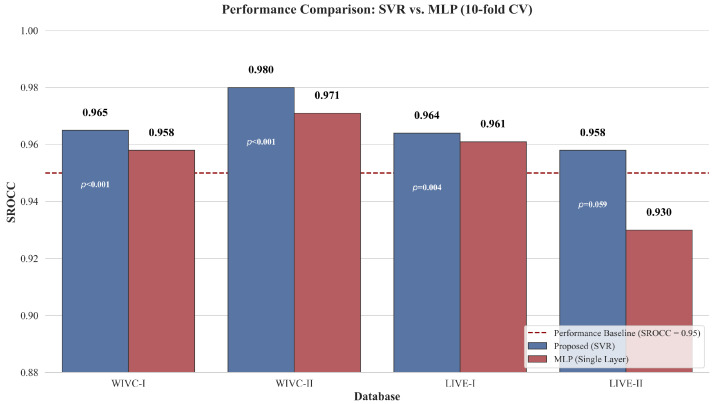
Statistical significance analysis between the proposed SVR head and the MLP head (Single-Layer) across 10 independent cross-validation runs. The error bars represent the standard deviation. The *p*-values (calculated via paired *t*-test) indicate that SVR achieves statistically significant superiority (p<0.06) on most datasets, particularly in reducing prediction variance.

Table 8 and Figure 6 compare the efficacy of different regression heads across the WIVC-II and LIVE-II datasets. The results demonstrate that SVR consistently surpasses both single layer and multi layer MLP architectures. This advantage is particularly evident in the RMSE on LIVE-II, where SVR reduces the error from 3.51 to 2.75, representing a significant gain in prediction stability. By effectively mapping high dimensional features to quality scores with fewer trainable parameters, the SVR head maintains superior generalization even when data is limited.

Complementing this analysis, Figure 6 visualizes the statistical trend across all four benchmark datasets. For clarity, the figure focuses on the comparison between SVR and the representative Single-layer MLP, further corroborating the consistent superiority of the SVR approach. This performance divergence is largely attributed to the limited scale of existing stereoscopic datasets. Neural networks typically require large-scale data to converge to a generalized optimum; on limited SIQA data, they are prone to overfitting despite heavy regularization. In contrast, SVR relies on the principle of structural risk minimization, which provides a more robust mapping from the high-dimensional feature space to quality scores, thereby mitigating the volatility often observed in pure neural regression.

#### 4.6.2. Ablation Study on Backbone Architectures

The performance of NR-SIQA models is inherently tied to the representational power of the extracted features. In this study, we focus on the intermediate-level outputs for all candidate backbones. This decision is grounded in the well-established observation in the IQA field that deep semantic features (near the output layers) often lack the spatial and structural fidelity required to characterize subtle image distortions [44]. While low-level features capture basic edges, intermediate-level features achieve an optimal balance between abstract semantics and perceptual details. Our extensive preliminary experiments across various backbone architectures further confirm that features from intermediate stages yield the highest correlation with human subjective scores in IQA tasks.

To provide a rigorous baseline, the SwinT (Stage 2, 384-D) is evaluated against two representative CNN-based architectures: VGG16 [55] (Pool4, 512-D) and ResNet50 [56] (Stage 4, 2048-D). Following the aforementioned protocol, all candidate backbones are assessed within a unified framework to ensure an unbiased comparison of their representational capacities. Specifically, the feature maps extracted from the particular stages or layers of the left and right views are converted into compact feature vectors through GAP. These monocular feature vectors are then fused via simple 0.5-weight averaging to form the input for the regression stage. For quality regression, we employ a SVR with a radial basis function kernel, where the optimal hyperparameters for each model are determined by an exhaustive grid search using 3-fold cross-validation on the training set. To ensure the statistical reliability of the performance metrics, the final SROCC, PLCC, and RMSE values are derived from the median of 10 independent iterations of 10-fold random split tests.

The results, summarized in Table 9, reveal that while classical CNNs provide competitive baselines, the Swin Transformer achieves a superior balance between representational efficiency and perceptual correlation. This justifies its selection as the foundational backbone for the proposed MSCE model. It is worth noting that while Stage 2 is utilized here as the representative benchmark for backbone comparison, the proposed framework is designed to leverage hierarchical features across multiple stages. The detailed investigation for the specific stage configuration are presented in the subsequent subsection.

#### 4.6.3. Ablation Study on Feature Extraction Stages

**Figure 7:**
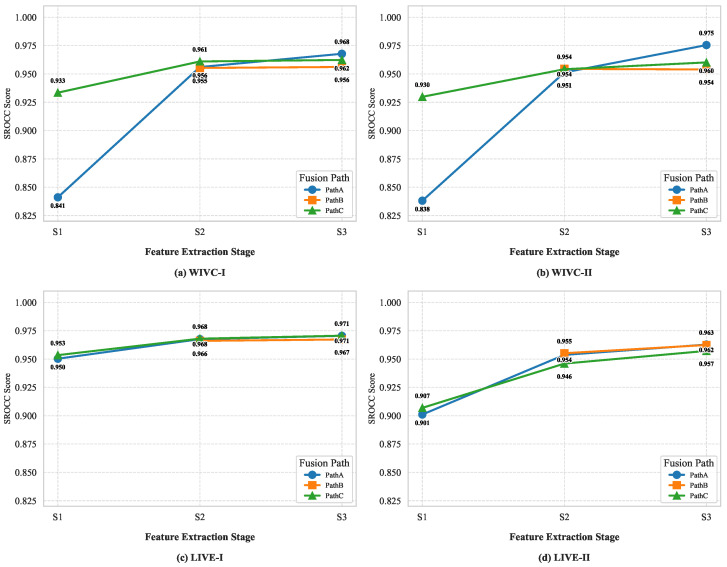
Performance comparison (SROCC) of different fusion paths across multiple feature extraction stages. The results validate that while Path A benefits from deeper features, the ASP-driven Paths B and C achieve optimal efficacy at Stage 2.

Following the selection of SwinT as the backbone, we further determine the optimal hierarchical levels for feature extraction. To investigate the interplay between feature hierarchy and fusion strategies, we conducted a comprehensive path-stage ablation study. We independently assessed the performance of each pathway (Path A, B, and C) when supplied with features from three hierarchical levels (Stage 1 to Stage 3) of the SwinT backbone, denoted as S1, S2, and S3. This design decouples the influence of feature depth from the inherent fusion logic. The results, illustrated in Figure 7, yield critical insights that guide our final framework configuration.

Observations from Figure 7 indicate that the pathways utilizing the ASP strategy (Path B and Path C) achieve their peak performance at Stage 2, but suffer from a noticeable performance regression at Stage 3. This behavior is intrinsically linked to the Competition-Aware Reinforcement mechanism inherent in ASP. Designed to simulate binocular rivalry, ASP employs aggressive nonlinear modulation to deterministically reinforce dominant features. While this strategy is highly effective on the mid-level features of Stage 2, where spatial structural details are well-preserved, it becomes detrimental when applied to the highly abstract semantic features of Stage 3. At such deep layers, the feature representations are semantically compressed; consequently, the aggressive reinforcement tends to be over amplified, causing semantic distortion rather than quality enhancement. In essence, applying strong competitive regulation to deep semantic representations disrupts their coherence, thereby degrading the model’s ability to assess perceptual quality.

In contrast to the dynamic nature of ASP, Path A employs a robust global prior (entropy-based baseline weights) without nonlinear reinforcement. Consequently, it exhibits a monotonic improvement as the network deepens, peaking at Stage 3. This suggests that the milder, linear integration strategy of Path A synergizes well with the rich global semantic context and larger receptive fields provided by deeper layers, allowing it to serve as a stable statistical anchor.

Although Stage 3 offers a marginal performance gain for the baseline Path A, it induces a fundamental conflict with the core ASP mechanism in Paths B and C by causing over-modulation of semantic features. Moreover, shifting to Stage 3 would double the feature dimensions from 384 to 768, incurring significant computational overhead. Therefore, to ensure the ASP strategy operates within its optimal modulation range we unify the feature extraction at Stage 2 for all pathways. This strategic configuration strikes a balance between the sharpen-then-smooth logic and feature representational integrity, maintaining a compact and efficient framework.

#### 4.6.4. Path-Wise Analysis of Hierarchical Features

The HCF module processes binocular information through three parallel pathways, each associated with a distinct abstraction level of the Swin Transformer backbone. This design reflects the multi-scale nature of human visual perception. To verify the individual contribution of these stages, we conduct a path-wise analysis by evaluating each path (Path A, B, and C) independently under the guidance of the ASP weight mechanism.

The results presented in Table 10 indicate that the performance gains are derived from synergistic interactions among stages rather than simple feature concatenation. Path A serves as the essential statistical foundation, and configurations incorporating this early stage pathway consistently outperform counterparts relying exclusively on deeper representations. This observation confirms that low level texture integrity provides a necessary grounding for the non linear decisions made in deeper stages. Path B and Path C contribute complementary mid level structural and high level semantic information, respectively. The complete configuration achieves global optimality by balancing these complementary cues, yielding the most consistent generalization across all tested datasets.

#### 4.6.5. Evaluation of Adaptive Selective Propagation and Design Optimality

Building upon the hierarchical HCF structure, we conduct a comprehensive evaluation to verify the effectiveness of the ASP strategy and the optimality of the weight matching design. The implementation of the ASP scheme is strictly governed by the functional positioning of hierarchical features. Specifically, low-level features in Path A remain close to raw pixels and thus require weights driven primarily by data statistics. Mid-level features in Path B capture structural degradation, benefiting from modulation driven by specific tasks, while high-level features in Path C represent abstract semantics where the maintenance of consistency is prioritized.

To demonstrate that the performance improvements are inherent to this architectural design rather than being dependent on specific hyperparameter settings, we compare seven distinct strategies across four datasets. The first group consists of homogeneous weighting schemes where all three pathways share the same modulation. These include a constant 0.5 weight (hereafter referred to as Equal), global entropy weights (Entropy), and uniform sharpening or smoothing modulations applied to all paths (Enhanced and Smoothed, respectively). The second group examines heterogeneous matching sequences to verify the specific alignment of features and weights. This group includes Reversed, which assigns smoothing to Path A and the entropy baseline to Path C, and Disordered, which assigns enhancement to Path A and the entropy baseline to Path B.

The results presented in Table 11 indicate that the proposed ASP consistently yields superior performance, particularly on the challenging WIVC-II dataset. While the performance margin between different matching sequences remains narrow on certain benchmarks, the ASP design exhibits exceptional stability across diverse datasets. Rather than pursuing a single optimal weight for a specific dataset, which might lead to empirical overfitting, we prioritize a robust scheme that aligns with the theoretical framework of hierarchical feature abstraction. This strategy ensures that the MSCE framework remains generalized across various stereoscopic distortion scenarios.

To ensure a fair and rigorous comparison, the performance results of the various strategies reported in Table 11 are obtained under identical SVR parameter settings. This uniform evaluation framework ensures that the observed differences in performance are strictly attributable to the architectural designs rather than incidental hyperparameter tuning. Building upon this fair baseline, we further conduct a sensitivity analysis to evaluate the stability of the proposed MSCE framework across diverse regression conditions. For this purpose, we define three representative configurations to cover the typical spectrum of the SVR hypothesis space. These settings range from a conservative configuration $C=256,\;\gamma_{\mathrm{svr}}=0.0019,\;\epsilon =0.5$, which prioritizes structural risk minimization and generalization, to an aggressive setting $C=1024,\;\gamma_{\mathrm{svr}}=0.0078,\;\epsilon =2.0$, which allows for higher complexity to capture finer data variations. A moderate baseline $C=512,\;\gamma_{\mathrm{svr}}=0.0039,\;\epsilon =1.0$ is also included to represent the standard operational state of the model. By testing across these distinct regimes of penalty costs and kernel scales, we can effectively assess the robustness of the hierarchical matching design.

**Figure 8:**
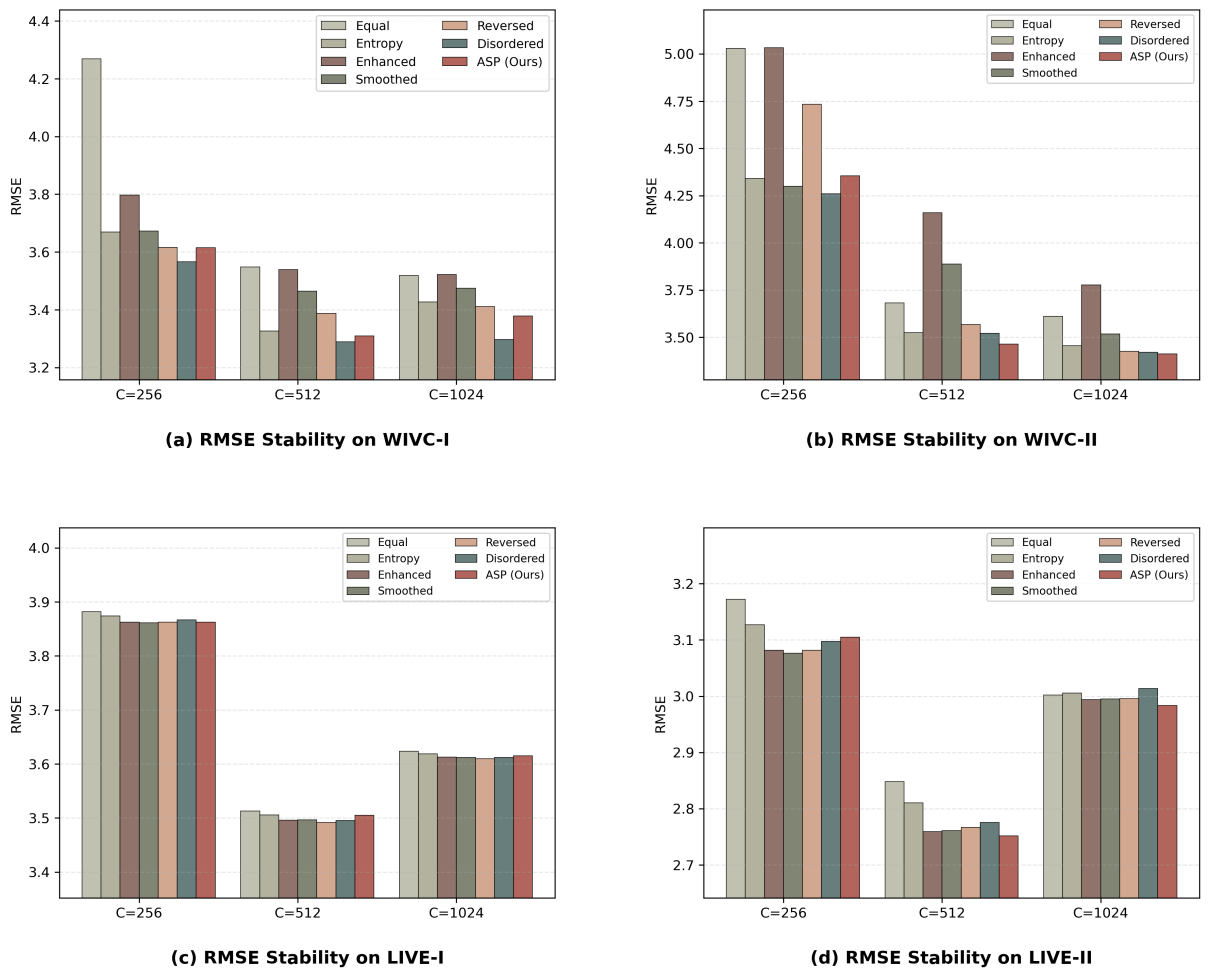
Robustness analysis of various matching strategies across different SVR parameter configurations on four benchmarks: (**a**) WIVC-I, (**b**) WIVC-II, (**c**) LIVE-I, and (**d**) LIVE-II. Each subfigure illustrates the RMSE performance under Conservative (C=256), Moderate (C=512), and Aggressive (C=1024) settings, demonstrating the architectural stability and optimality of the proposed ASP design.

As illustrated in Figure 8, the RMSE performance remains remarkably consistent across these diverse configurations. This observation confirms that the performance gains are an inherent property of the structural design rather than a consequence of specific parameter optimization. It is worth noting that while alternative matching sequences such as Disordered achieve slightly lower error rates on specific benchmarks like WIVC-I or LIVE-I under certain parameters, such empirical gains often lack theoretical consistency and universal stability across diverse datasets. The consistent superiority of our hierarchical alignment, which progresses from low-level texture to high-level semantics, indicates that following the natural processing stages of the human visual system provides a more robust and interpretable solution for stereoscopic quality assessment. By prioritizing a design that aligns with biological perception rather than pursuing the highest numerical score on a single dataset, the ASP strategy ensures strong generalization across varying distortion types. Even when each strategy is evaluated under its own optimal parameter set, the ASP design maintains a steady lead in overall performance, which validates both the rationality and the optimality of the proposed architectural choice.

#### 4.6.6. Hyperparameter Sensitivity Analysis of the ASP Strategy

To investigate the impact of the reinforcement boundaries ($E_{\min},E_{\max}$) and the adaptivity factor γ, we conducted a comprehensive sensitivity analysis. As summarized in Table 12 and Table 13, the experimental regimes are categorized by their reinforcement intensity: Weak and Normal represent conservative gain control; Static denotes a configuration with a fixed reinforcement boundary ($E_{\min}=E_{\max}$); while Moderate to Saturated explore progressively aggressive nonlinear selectivity driven by the rivalry mechanism.

As shown in Table 12 and Table 13, the transition from Weak to Normal highlights the decisive role of $E_{\max}$ in modulating asymmetric distortions, where performance on WIVC-II and LIVE-II improves consistently with higher boundaries. However, the Static strategy reveals that a fixed boundary lacks the flexibility to handle varying binocular disparities. While the Saturated pushes the RMSE on WIVC-II to its minimum (3.370), it simultaneously induces a performance regression on the symmetric-dominant LIVE-I dataset (RMSE 3.527). This indicates that inflexible, high-intensity reinforcement over-sensitizes the model, leading to an unwanted “asymmetric bias” even in balanced states.

To resolve this conflict, the adaptivity factor γ is introduced as a stability regulator to transition from static gain control to entropy-guided modulation. Based on the performance across all benchmarks, the Proposed configuration ($E_{\min}=2.0,\;E_{\max}=4.0,\;\gamma =0.6$) achieves the most calibrated balance. Unlike the Saturated regime, which suffers from a significant 0.6% RMSE increase on LIVE-I, the proposed setup applies aggressive nonlinear propagation only when binocular disparity is substantial, while reverting to conservative fusion for symmetric cases. Ultimately, this configuration is selected as the optimal setup; it successfully leverages the benefits of competitive selectivity for binocular rivalry while maintaining high fidelity for binocular fusion, demonstrating superior generalization across diverse stereoscopic scenarios.

While our analysis indicates that $E_{\min}\geq 2.0$ provides more pronounced nonlinear reinforcement for binocular rivalry, we conducted a comprehensive sensitivity analysis covering a wider parameter range ($E_{\min}$ from 1.10 to 4.00) to validate the robustness of the proposed configuration.

### 4.7. Failure Case Analysis

**Figure 9:**
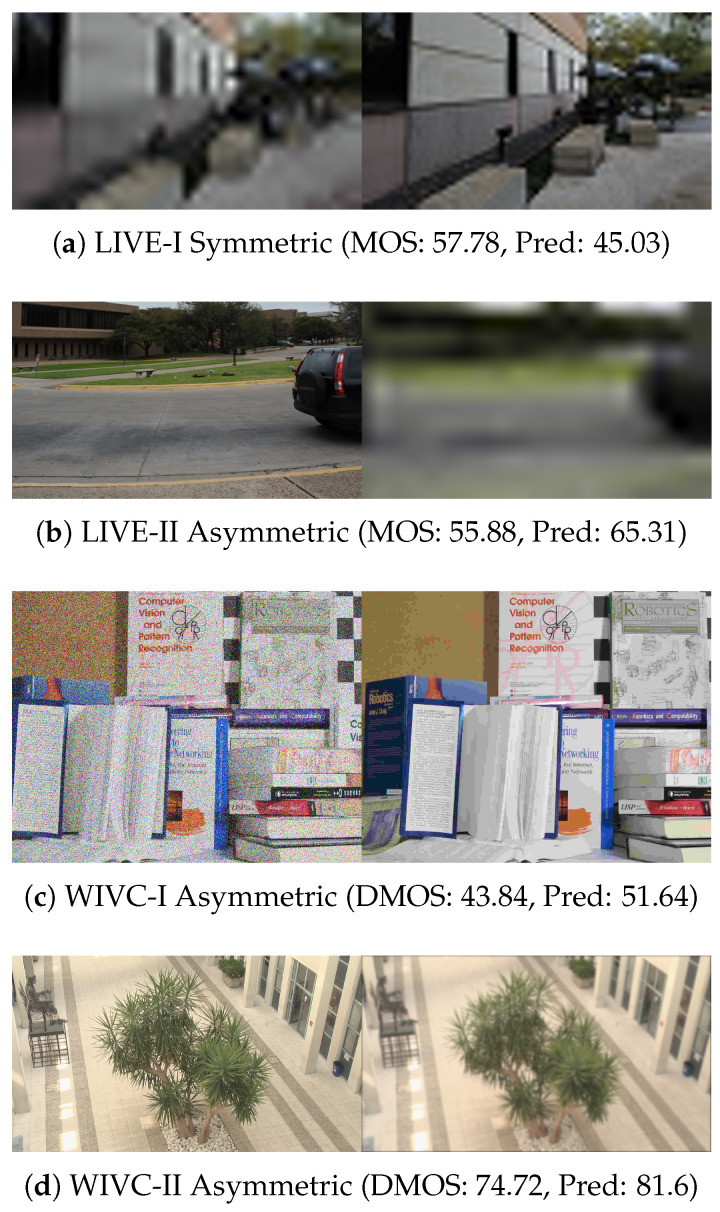
Visualization of representative failure cases across four datasets. (**a**) LIVE-I (FF): Severe structural corruption leads to feature ambiguity. (**b**) LIVE-II (Ref-GB): Extreme asymmetry poses challenges for the rivalry suppression mechanism. (**c**) WIVC-I (WN-JP2K) and (**d**) WIVC-II (Ref-JP2K): High-frequency textures in high-resolution images may mask artifacts for human observers, causing the model to over-penalize quality (Texture Masking). The specific MOS and predicted scores are provided to illustrate the deviation.

To objectively assess the limitations of the MSCE framework, we selected one representative sample exhibiting a prediction deviation beyond the RMSE margin from each of the four benchmark datasets, as presented in Figure 9. These cases reveal three primary challenges in current stereoscopic quality modeling. For the LIVE I sample shown in Figure 9a, severe structural degradation and low spatial resolution compromise the global integrity of the image, causing the backbone network to extract incoherent features that lead to inaccurate quality estimates. In the case of extreme asymmetric rivalry exemplified by the LIVE II sample in Figure 9b, the model occasionally assigns residual weight to the blurred view despite the presence of a sharp counterpart. This suggests that the dominance established during the competitive reinforcement phase can be partially diluted by subsequent binocular smoothing, preventing the model from fully converging to the “winner-take-all” percept. Furthermore, for the WIVC I and WIVC II samples with high spatial resolution and complex textures in Figure 9c,d, the model tends to overestimate the severity of artifacts. While the human visual system masks subtle distortions in texture-rich regions, the model relies on pixel-level contrast and remains sensitive to artifacts that are perceptually masked. These conclusions underscore the necessity of integrating texture sensitivity priors to better emulate the masking effects of human perception in future research.

### 4.8. Visualization of Performance

**Figure 10:**
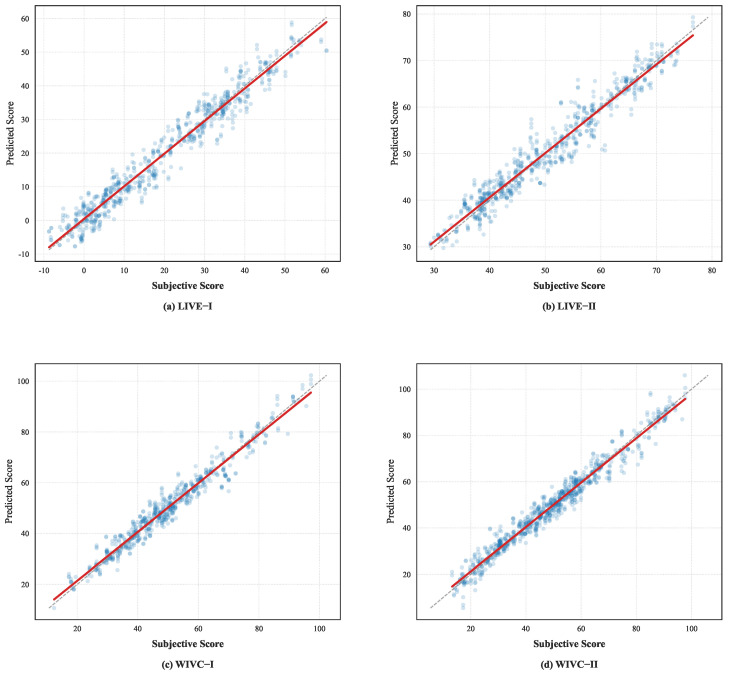
Scatter plots of predicted quality scores vs. subjective scores. The data points represent the aggregated results from 10 random train–test splits for (**a**) LIVE-I, (**b**) LIVE-II, (**c**) WIVC-I, and (**d**) WIVC-II. The tight clustering along the diagonal red dashed line demonstrates the high consistency between the algorithmic predictions and human subjective judgments.

To further evaluate the prediction stability and fitting accuracy of the proposed framework, we provide scatter plots of the predicted scores versus the subjective scores across four benchmark datasets in Figure 10. For clear visualization, the train–test splitting procedure was repeated five times using different random seeds, and the scatter plots aggregate the test samples from these independent runs. The SROCC, PLCC, and RMSE values annotated in Figure 10 correspond to the averaged performance across these splits. In Figure 10, the red dashed line represents the ideal linear relationship (y=x). Across all datasets, the scatter points cluster closely around the diagonal, indicating strong linear correlations and low dispersion between the predicted scores and subjective human ratings. On the challenging WIVC-II dataset, which contains complex mixed-type asymmetric distortions, the model still exhibits high prediction fidelity, with no significant outliers. This confirms that the ASP module effectively balances inter-view contributions. Overall, these visual results demonstrate that the proposed method delivers reliable and consistent quality assessment across diverse stereoscopic content and distortion types.

### 4.9. Model Efficiency and Complexity

To assess the practical feasibility of the MSCE framework, we analyze its computational overhead in terms of theoretical complexity and architectural efficiency. Let 𝒪(SwinT) denote the cost of a single forward pass through the backbone. Given the weight-sharing strategy, the primary feature extraction cost is defined as $C_{\mathrm{extract}}=2\times 𝒪\left(\mathrm{SwinT}\right)$. The additional computational cost introduced by the ASP strategy and fusion pathways is negligible. Since the backbone parameters are frozen, the optimization process is restricted to the lightweight ASP modules and the regressor. This design significantly reduces the training overhead compared to end-to-end fine-tuning architectures. Furthermore, by decoupling binocular interactions into a deterministic weighting and regression process, the framework avoids the high-dimensional instability and overfitting risks associated with full-parameter optimization. Such architectural efficiency ensures stable prediction performance while facilitating the deployment of MSCE in real-time stereoscopic quality monitoring systems.

## 5. Discussion

### 5.1. Evolution of Assessment Paradigms and Biological Foundations

The design of the MSCE framework reflects the broader paradigm shift in quality assessment, moving from local modeling via convolutional neural networks to global perceptual assessment driven by Transformers. As noted in recent surveys [30], architectures using the Transformer model offer superior capacity for capturing long range dependencies essential for perceptual modeling. Our work extends this scope by embedding biological mechanisms of binocular rivalry within a hierarchical Swin Transformer backbone. Looking forward, this rivalry conscious design could be further enhanced by exploring attention mechanisms across views to simulate the dynamic temporal fluctuations of binocular suppression. Such architectures have the potential to adapt to increasingly complex immersive environments, where the interaction between ocular disparity and temporal consistency is critical for perceptual quality.

### 5.2. Perceptual and Practical Implications

The experimental outcomes validate that binocular perception operates as a progressive integration process rather than a static summation. Unlike methods using fixed weighting, the proposed ASP strategy effectively captures this dynamic nature. Sensitivity analysis shows that the model reinforces the leading view as binocular disparity increases. This nonlinear reinforcement behavior aligns with Stevens’ Power Law, indicating that the model effectively simulates the discriminative selection mechanism of the primary visual cortex. Furthermore, the evolution of weights across stages reveals a stable dynamic: intermediate stages sharpen features to resolve competition between views, while deeper semantic stages smooth them to ensure coherent global integration. Consequently, the HCF module functions similarly to ensemble learning, pairing the representational capacity of modern backbones with biologically inspired fusion strategies.

The MSCE framework demonstrates strong generalization across diverse benchmarks, suggesting that the model captures intrinsic perceptual features rather than dataset specific biases. This transferability is particularly valuable in practical applications with scarce annotated stereoscopic data. For example, in virtual reality and mixed reality environments where binocular discomfort can cause motion sickness, the model can be integrated into rendering engines to provide real-time quality feedback. In robotic assisted stereoscopic surgery, the high precision of the framework ensures that the visual feed remains perceptually reliable. Ultimately, by providing a metric centered on human perception, our work supports the development of more immersive stereoscopic systems and establishes a foundation for future applications in video quality assessment.

### 5.3. Limitations and Strategic Outlook

Despite the competitive performance achieved by the MSCE framework, several limitations warrant further discussion. A primary constraint involves the reliance on kernel Support Vector Regression (SVR). While SVR ensures stability in small sample regimes typical of current stereoscopic datasets, its computational complexity increases quadratically with the number of training samples. This characteristic poses a scalability challenge for large scale applications, necessitating the exploration of efficient approximation methods or alternative regression heads as data volumes grow. Beyond computational complexity, the current ASP strategy primarily models global inter-view competition, while local rivalry is captured only implicitly through the self-attention mechanism. Future research aims to introduce spatial attention mechanisms to precisely localize competing regions within a stereo pair, thereby providing a more granular analysis of local binocular interactions. Parallel to this, the integration of explicit depth geometry priors remains an essential objective to more accurately assess three dimensional structural distortions. Looking forward, extending the competitive fusion principle to stereoscopic video quality assessment represents a significant strategic challenge. We envision evolving the ASP strategy into a spatial and temporal propagation mechanism, potentially integrating recurrent units to track the winner take all process across both ocular and temporal domains. This extension is vital for capturing dynamic artifacts, such as temporal inconsistency and motion induced suppression, which are prevalent in immersive visual media. By addressing these computational and perceptual constraints, the proposed framework establishes a robust foundation for next generation stereoscopic systems.

## 6. Conclusions

This paper presents the MSCE framework for no-reference stereoscopic image quality assessment, which integrates the visual mechanisms of binocular rivalry and fusion within a hierarchical Transformer architecture. The core innovations, namely the ASP strategy and HCF module, introduce a stage-wise scheme to dynamically regulate the transition between binocular rivalry and fusion. By applying nonlinear gain control to reinforce the perceived dominance of the leading view, the framework effectively models the progressive nature of human binocular integration. Experimental results on four benchmark datasets demonstrate that MSCE achieves state-of-the-art performance, particularly in challenging scenarios involving asymmetric distortions. Ablation studies validate the critical roles of competitive selectivity and the ensemble-like integration of multi-scale features. Furthermore, the strong generalization of the framework across diverse datasets highlights its potential for practical applications in 3D visual signal processing. In summary, this work advances the development of perceptually faithful computational models for SIQA by embedding a dynamic binocular rivalry mechanism into a modern hierarchical backbone. Future research will explore the introduction of spatial attention mechanisms to precisely localize competing regions within a stereo pair, thereby providing a more granular analysis of local binocular interactions. Additionally, designing region-specific dynamic weight adjustment strategies could further enhance the model’s capacity to handle complex non-uniform distortions. Beyond static images, extending this architecture to account for temporal consistency in stereoscopic videos and exploring depth geometry priors remain important directions for further improving the assessment of binocular perception.

## Figures and Tables

**Table 1:** Weight evolution across stages for representative distortion samples (Scene: CraftLoom).

Distortion Type	Baseline $\alpha_{L}$	Stage 1 $w_{L}^{\left(1\right)}$	Stage 2 $w_{L}^{\left(2\right)}$	ASP Effect
Symmetric (G2–G2)	0.5005	0.5020	0.5020	Integration
Asymmetric (Re–J2)	0.4021	0.1821	0.1933	Dominance
Asymmetric (G4–G1)	0.5185	0.5688	0.5683	Sharpening
Asymmetric (W2–J1)	0.4628	0.3667	0.3685	Sharpening
Asymmetric (W2–W4)	0.4847	0.4425	0.4428	Sharpening

**Table 2:** Performance comparison on WIVC-I and WIVC-II datasets. The top performance is bolded. The superscripts “*” indicate that the difference between the proposed MSCE and the second-best method is statistically significant (p<0.05) based on a paired *t*-test.

	WIVC-I			WIVC-II		
Method	SROCC	PLCC	RMSE	SROCC	PLCC	RMSE
ChenFR [13]	0.509	0.674	11.623	0.444	0.569	15.740
IDWSSIM [10]	0.950	0.961	4.359	0.944	0.953	5.781
BRISQUE [50]	0.935	0.950	4.951	0.933	0.945	6.294
ChenNR [14]	0.911	0.926	6.232	0.884	0.882	8.961
Shen [38]	0.906	0.919	–	0.852	0.863	–
Si [6]	*0.963*	0.963	3.635	0.966	*0.971*	*4.161*
Sim [37]	*0.963*	0.957	4.192	0.968	0.970	4.598
Liu [54]	0.928	0.945	5.268	0.901	0.913	7.658
Chang [20]	0.953	0.950	*3.343*	0.967	0.952	4.289
X-Net [40]	0.960	*0.968*	4.070	*0.969*	0.975	4.592
Li [21]	0.968	0.972	3.326	0.970	*0.971*	3.536
Proposed (MSCE)	**0.971** *	**0.978** *	**3.309** *	**0.983** *	**0.984** *	**3.465** *

Note: The best, second-best, and third-best performances are highlighted in bold, underlined, and italicized, respectively.

**Table 3:** Performance comparison on LIVE-I and LIVE-II datasets. The superscripts “*” indicate statistical significance (p<0.05) against the second-best deep learning method.

	LIVE-I			LIVE-II		
Method	SROCC	PLCC	RMSE	SROCC	PLCC	RMSE
ChenFR [13]	0.916	0.834	6.268	0.901	0.778	4.746
IDWSSIM [10]	–	–	–	0.916	0.919	–
BRISQUE [50]	0.949	0.957	4.827	0.938	0.943	3.840
ChenNR [14]	0.933	0.922	–	0.880	0.880	5.102
Shen [38]	0.952	0.962	4.493	0.940	0.950	3.546
StereoQA [16]	–	–	–	0.947	0.957	3.270
Si [6]	0.966	**0.977**	–	0.953	**0.972**	–
Sim [37]	0.968	0.962	3.944	*0.961*	0.955	3.042
Chang [20]	*0.970*	*0.971*	–	*0.961*	0.956	–
X-Net [40]	0.971	0.976	4.103	**0.969**	0.961	5.029
Li [21]	0.973	**0.977**	*4.058*	*0.961*	*0.963*	*3.061*
Proposed (MSCE)	**0.975** *	**0.977**	**3.505** *	0.968	0.970	**2.752** *

Note: The best, second-best, and third-best performances are highlighted in bold, underlined, and italicized, respectively.

**Table 4:** Performance comparison (SROCC and PLCC) for individual distortion types on the LIVE-I dataset.

	SROCC					PLCC				
Method	JP2K	JPEG	WN	GB	FF	JP2K	JPEG	WN	GB	FF
Liu [54]	0.888	0.785	0.951	0.917	0.821	0.938	0.810	0.966	0.956	0.855
Shen [38]	*0.958*	0.801	0.971	0.965	0.883	0.937	0.779	0.959	0.921	0.851
Wang [40]	0.936	0.841	0.952	0.900	0.856	*0.965*	0.875	*0.975*	0.966	0.910
Chang [20]	0.960	*0.904*	*0.970*	*0.983*	0.960	**0.988**	0.917	0.972	0.993	*0.943*
X-Net [40]	**0.974**	0.907	0.932	0.993	**0.968**	0.986	*0.898*	0.988	*0.983*	**0.977**
Li [21]	0.951	**0.924**	**0.997**	**0.994**	0.910	*0.965*	**0.935**	**0.996**	**0.996**	0.958
Proposed	0.941	0.869	0.971	0.927	*0.925*	0.950	0.861	0.973	0.951	0.929

Note: The best, second-best, and third-best performances are highlighted in bold, underlined, and italicized, respectively.

**Table 5:** Performance comparison (SROCC and PLCC) for individual distortion types on the LIVE-II dataset.

	SROCC					PLCC				
Method	JP2K	JPEG	WN	GB	FF	JP2K	JPEG	WN	GB	FF
Liu [54]	0.909	0.825	0.946	0.936	0.938	0.936	0.867	0.969	0.987	0.959
Shen [38]	0.946	**0.903**	0.831	0.912	0.939	0.974	**0.922**	0.858	0.977	0.949
Wang [40]	0.916	*0.876*	*0.961*	*0.965*	0.952	0.943	0.921	0.978	*0.994*	*0.967*
Chang [20]	**0.961**	0.866	0.934	0.881	0.946	**0.981**	0.905	0.965	0.997	**0.969**
X-Net [40]	0.927	0.893	0.955	0.959	**0.974**	0.941	0.903	*0.976*	0.991	0.936
Li [21]	*0.942*	0.874	**0.984**	**0.980**	*0.959*	0.936	*0.907*	**0.999**	**0.997**	0.961
Proposed	0.946	0.862	0.968	0.969	0.966	*0.952*	0.891	0.974	0.993	0.968

Note: The best, second-best, and third-best performances are highlighted in bold, underlined, and italicized, respectively.

**Table 6:** Performance evaluation (SROCC, PLCC, and RMSE) of the proposed method on Symmetric and Asymmetric subsets. For internal comparison, four decimal places are preserved.

Dataset	Overall			Symmetric			Asymmetric		
	SROCC	PLCC	RMSE	SROCC	PLCC	RMSE	SROCC	PLCC	RMSE
WIVC-I	0.9708	0.9781	3.3093	0.9712	0.9785	3.2845	0.9698	0.9775	3.3520
WIVC-II	0.9827	0.9839	3.4645	0.9832	0.9845	3.3982	0.9824	0.9836	3.4912
LIVE-II	0.9681	0.9702	2.7518	0.9692	0.9721	2.6845	0.9675	0.9693	2.7830

**Table 7:** Cross-dataset validation results (SROCC/PLCC). The notation Test/Train indicates that the model is trained on the second dataset and evaluated on the first.

Method	WIVC-II/I	WIVC-I/II	LIVE-II/I	LIVE-I/II
BRISQUE [50]	0.841/0.870	0.853/0.832	0.732/0.768	0.657/0.671
Sim [37]	0.755/0.786	0.903/0.914	0.770/0.804	0.896/0.908
Chang [20]	0.912/0.922	**0.949**/**0.936**	*0.807*/*0.847*	**0.906**/**0.910**
Li [21]	**0.914**/**0.909**	0.931/0.928	**0.92**/**0.919**	0.855/0.838
Proposed	*0.881*/*0.892*	*0.922*/*0.930*	0.818/0.831	*0.885*/*0.868*

Note: The best, second-best, and third-best performances are highlighted in bold, underlined, and italicized, respectively.

**Table 8:** Comparison between SVR and MLP regression heads. The best performance is bolded. SVR consistently achieves lower RMSE, indicating higher stability. The best performance is highlighted in bold.

Regression Head	WIVC-II			LIVE-II		
	SROCC	PLCC	RMSE	SROCC	PLCC	RMSE
MLP (Single-Layer)	**0.9829**	0.9824	3.748	0.9571	0.9537	3.511
MLP (Double-Layer)	0.9808	0.9820	3.767	0.9564	0.9552	3.533
Proposed (SVR)	0.9828	**0.9835**	**3.465**	**0.9680**	**0.9689**	**2.752**

**Table 9:** Performance comparison of different architectural variants under a consistent SVR configuration.

Backbone	Dim	WIVC-I			WIVC-II			LIVE-I			LIVE-II		
		SROCC	PLCC	RMSE	SROCC	PLCC	RMSE	SROCC	PLCC	RMSE	SROCC	PLCC	RMSE
VGG16	512	0.9567	0.9534	4.8689	0.9658	0.9599	5.1702	0.9773	0.9720	3.5236	0.9531	0.9438	3.4582
ResNet50	2048	0.9397	0.9195	5.5431	0.9361	0.9404	6.9425	0.9740	0.9642	3.8547	0.9473	0.9422	3.6386
SwinT	384	0.9376	0.9534	4.7610	0.9539	0.9598	5.4519	0.9700	0.9717	3.8738	0.9510	0.9552	3.3451

**Table 10:** Ablation results for different path configurations across four datasets. The best, second-best, and third-best results are highlighted in bold, underline, and italic, respectively.

Path	WIVC-I			WIVC-II			LIVE-I			LIVE-II		
	SROCC	PLCC	RMSE	SROCC	PLCC	RMSE	SROCC	PLCC	RMSE	SROCC	PLCC	RMSE
Path A	0.9674	0.9735	3.6114	0.9438	0.9552	5.7796	0.9708	0.9722	3.8424	0.9575	0.9606	3.1455
Path B	0.9597	0.9642	4.1917	*0.9601*	0.9567	*5.5888*	0.9701	0.9721	3.8484	0.9557	0.9586	3.2246
Path C	0.9663	0.9708	3.7743	0.9576	0.9529	5.8570	0.9697	0.9712	3.9102	*0.9609*	*0.9631*	*3.0396*
Path AB	*0.9694*	0.9753	3.4768	0.9827	**0.9840**	**3.4098**	*0.9718*	0.9740	3.7184	0.9624	0.9652	2.9561
Path AC	0.9696	*0.9751*	*3.4931*	0.9793	*0.9816*	*3.6628*	0.9719	*0.9738*	*3.7353*	0.9608	0.9635	3.0235
Path BC	0.9666	0.9713	3.7480	*0.9608*	0.9546	5.7447	0.9705	0.9716	3.8818	0.9571	0.9601	3.1628
Full ABC	**0.9706**	**0.9776**	**3.3093**	**0.9828**	0.9835	3.4645	**0.9745**	**0.9769**	**3.5052**	**0.9680**	**0.9698**	**2.7518**

**Table 11:** Comparison of strategies for matching weights to hierarchical paths: best performance achieved across three distinct SVR parameter configurations. The best and second-best results are highlighted in bold and underline, respectively.

Strategy	WIVC-I			WIVC-II			LIVE-I			LIVE-II		
	SROCC	PLCC	RMSE	SROCC	PLCC	RMSE	SROCC	PLCC	RMSE	SROCC	PLCC	RMSE
Equal	0.9678	0.9747	3.5203	0.9797	0.9813	3.6826	0.9747	0.9770	3.4983	0.9668	0.9686	2.8050
Entropy	0.9705	0.9774	3.3266	0.9822	0.9829	3.5240	0.9744	0.9769	3.5060	0.9666	0.9685	2.8103
Enhanced	0.9660	0.9744	3.5397	0.9766	0.9761	4.1595	0.9747	0.9770	**3.4963**	0.9677	0.9697	2.7595
Smoothed	0.9673	0.9755	3.4647	0.9798	0.9792	3.8877	0.9747	0.9770	3.4966	0.9676	0.9696	2.7609
Reversed	0.9693	0.9766	3.3873	0.9824	0.9825	3.5672	**0.9748**	**0.9771**	3.4921	0.9678	0.9695	2.7666
Disordered	**0.9706**	**0.9779**	**3.2896**	0.9823	0.9829	3.5218	0.9744	0.9770	3.4955	0.9674	0.9693	2.7756
ASP (Proposed)	**0.9706**	0.9776	3.3093	**0.9828**	**0.9835**	**3.4645**	0.9745	0.9769	3.5052	**0.9680**	**0.9698**	**2.7518**

**Table 12:** Hyperparameter sensitivity analysis on WIVC datasets. Only the best performance for each metric is highlighted in bold to demonstrate the trade-off between symmetric and asymmetric scenarios.

Regime	Parameters			WIVC-I			WIVC-II		
	$E_{\min}$	$E_{\max}$	γ	SROCC	PLCC	RMSE	SROCC	PLCC	RMSE
Weak	1.10	1.20	0.3	0.9704	0.9776	3.315	0.9823	0.9831	3.508
Normal	1.30	1.80	0.3	**0.9709**	**0.9778**	**3.299**	0.9826	0.9834	3.475
Static	2.00	2.00	0.6	0.9704	0.9777	3.302	0.9826	0.9834	3.473
Moderate	2.00	4.00	0.8	0.9705	0.9775	3.321	0.9827	0.9837	3.446
Intense	2.00	4.00	1.0	0.9701	0.9774	3.330	0.9827	0.9839	3.423
Saturated	4.00	8.00	1.0	0.9706	0.9773	3.332	**0.9838**	**0.9844**	**3.370**
Proposed	**2.00**	**4.00**	**0.6**	0.9706	0.9776	3.309	0.9828	0.9835	3.465

**Table 13:** Hyperparameter sensitivity analysis on LIVE datasets. The Proposed configuration is chosen for its superior numerical stability on the symmetric-only LIVE-I dataset. The best performance for each metric is highlighted in bold.

Regime	Parameters			LIVE-I			LIVE-II		
	$E_{\min}$	$E_{\max}$	γ	SROCC	PLCC	RMSE	SROCC	PLCC	RMSE
Weak	1.10	1.20	0.3	0.9744	**0.9769**	3.506	0.9667	0.9686	2.807
Normal	1.30	1.80	0.3	0.9743	**0.9769**	3.507	0.9669	0.9688	2.799
Static	2.00	2.00	0.6	0.9744	**0.9769**	3.507	0.9673	0.9690	2.790
Moderate	2.00	4.00	0.8	**0.9746**	**0.9769**	**3.505**	0.9682	**0.9699**	2.751
Intense	2.00	4.00	1.0	**0.9746**	**0.9769**	**3.505**	0.9683	**0.9699**	**2.750**
Saturated	4.00	8.00	1.0	0.9745	0.9766	3.527	**0.9685**	**0.9699**	**2.750**
Proposed	**2.00**	**4.00**	**0.6**	0.9745	**0.9769**	**3.505**	0.9680	0.9698	2.752

## Data Availability

The original contributions presented in this study are included in the article. Further inquiries can be directed to the corresponding author.
